# Two Unusual Methylidenecyclopropane Glucosides from *Metaxya rostrata* C.Presl

**DOI:** 10.1002/hlca.201200111

**Published:** 2012-09-13

**Authors:** Kerstin P Kainz, Judith Virtbauer, Hanspeter Kählig, Vladimir Arion, Oliver Donath, Gottfried Reznicek, Werner Huber, Brigitte Marian, Liselotte Krenn

**Affiliations:** a Department of Pharmacognosy, University of ViennaAlthanstr. 14, AT-1090 Vienna; b Institute of Organic Chemistry, University of ViennaWähringerstr. 38, AT-1090 Vienna; c Institute of Inorganic Chemistry, University of ViennaWähringerstr. 42, AT-1090 Vienna; d Department of Structural Botany, University of ViennaRennweg 14, AT-1030 Vienna; e Institute of Cancer Research, Medical University of ViennaBorschkegasse 8a, AT-1090 Vienna

**Keywords:** *Metaxya rostrata*, Glycosides, Cyclopropanes, methylidene-, X-Ray crystallography

## Abstract

Two new natural compounds, (1*R*,2*E*)-2-(6-hydroxyhexylidene)cyclopropyl-*β*-D-glucopyranoside (**1**) and (6*E*)-6-[(2*R*)-2-(*β*-D-glucopyranosyloxy)cyclopropylidene]hexanoic acid (**2**), glucosides of a very rare methylidenecyclopropane alcohol, as well as two known glycosides of phenolic acids, namely 4-*O*-*β*-D-glucopyranosylcaffeic acid (**3**) and (*E*)-4-*O*-*β*-D-glucopyranosylcoumaric acid (**4**), and methyl *α*-fructofuranoside (**5**) were isolated for the first time from the rhizomes of the tree fern *Metaxya rostrata* C.Presl. The structures were elucidated on the basis of detailed spectroscopic data analysis, and the structure of 1 was additionally confirmed by X-ray crystal-structure analysis.

## Introduction

The tree fern *Metaxya rostrata* C.Presl (Metaxyaceae) is distributed widely in lowland rain forests of Central and South America, and is used in traditional medicine in Costa Rica against intestinal diseases such as ulcers or tumors. For the treatment of these diseases, a suspension of the dried rhizome of *Metaxya rostrata* in water is applied orally [1]. As tropical plants are a rich and promising source for new cytotoxic compounds [2], and no data were available on the chemical composition of this plant, the rhizomes of *Metaxya rostrata* were studied phytochemically. In our previous investigation, an aqueous extract of the rhizomes was fractionated. Within this earlier study, two cytotoxic proanthocyanidins (cinnamtannin B-1 and aesculitannin B), common sterols, and sugars had been isolated [1]. Further separation of other fractions from the aqueous extract which did not show significant cytotoxicity should give a more detailed insight into the chemical composition of this plant.

## Results and Discussion

**Figure 1:**
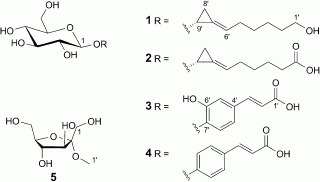
Structures of compounds 1–5

Rhizomes of *Metaxya rostrata* were extracted with hot water according to the ethnomedicinal procedure and fractionated. After vacuum liquid chromatography and repeated column chromatography on silica gel or *Sephadex LH-20*, two new compounds 1 and 2, two phenolic acid glycosides 3 and 4, and a methyl glycoside 5 have been isolated (Fig. 1).

The characterization of 1, a white crystalline substance, was achieved by means of mass spectrometry and extensive 1D- and 2D-NMR experiments. From the ESI mass spectra, a molecular weight of 318 Da was deduced. Using flow injection analysis (FIA), the mass spectrum in the negative-ion mode displayed the [*M* − H]^−^ ion peak at *m*/*z* 317, whereas in positive-ion mode not only the [*M*+H]+ ion peak at *m*/*z* 319, but also the [*M*+NH_4_]^+^ and [*M*+Na]+ peaks at *m*/*z* 336 and 341, respectively, were detected. Product ion peaks were recorded with MS/MS in both, the positive- and negative-ion mode at various collision energies. The [*M*+H]+ ion showed mainly fragmentation of the glycosidic bond to yield the Y^+^_0_ ion (*m*/*z* 157) and the Z^+^_0_ ion (*m*/*z* 139). The [*M* − H]^−^ ion produced the complementary C^−^_1_ and B^−^_1_ fragment ions (*m*/*z* 179 and 161, resp.), as well as the ^0,2^A^−^ and the ^0,3^A^−^ ions (*m*/*z* 119 and 89, resp.), resulting from cross-ring cleavage of the hexose. The HR-ESI-MS revealed a molecular weight of 318.3618 in accordance with the molecular formula C_15_H_25_O_7_.

**Figure 2:**
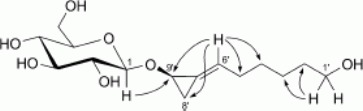
HMBCs of compound 2

In the NMR experiments, the sugar part was easily characterized as a β-glucopyranose. The aglycon showed mainly signals for CH_2_ groups in the aliphatic region, one C = C bond CH signal, and an additional CH signal at 4.18 ppm in the ^1^H-NMR. The ^13^C-NMR additionally showed the resonance of a quaternary olefinic Catom at 123.9 ppm. The structure of the aglycon could be identified as a 2-(6- hydroxyhexylidene)cyclopropanol moiety (for the structure, see Fig. 1), linked glycosidically with glucose *via* the alcohol attached to the cyclopropane ring. This result is based on the characteristic ^1^H- and ^13^C-NMR chemical shifts (Table 1) of the cyclopropane signals together with rather large ^1^H,^13^C one-bond coupling constants, namely 188.2 Hz for H^−^C(9’), and 163.9 and 161.0 Hz for the CH_2_ (8’), respectively [3]. The observed C = C stretching frequency of 1775 cm^−^_1_ in the IR spectrum of 1 is in accordance with published data of similar methylidenecyclopropane structures [4]. Numerous HMBCs established all the necessary connectivities, *e.g.*, the interglycosidic linkage by a cross-peak from H − C(1) to C(9’), or correlations within the aglycon from H − C(6’) to the C(9’), C(8’), C(5’), and C(4’), and from H − C(1’) to C(2’) and C(3’), respectively (Fig. 2). NOESY Cross-peaks from CH_2_(5’) to CH_2_ (8’) within the cyclopropanol revealed the geometry of the C = C bond. Attached to the C = C bond is a C_5_ aliphatic chain terminated by an alcohol function. From all data above, the structure of compound 1 was established as (2*E*)-2-(6-hydroxyhexylidene)cyclopropyl-β- d-glucopyranoside.

**Table 1:** ^1^H- (600.13 MHz) *and* ^13^C-NMR (150.92 MHz) Data of 1 and 2 in CD_3_OD (δ in ppm; *J* in Hz)

Position	1		2	
	(δ)(H)	(δ)(C)	(δ)(H)	(δ)(HC)
1	4.39 (*d, J* 7 = 7.9)	104.2	4.39 *(d, J* 7 = 7.9)	104.2
2	3.14 (*dd, J* 7 = 7.9, 9.2)	74.7	3.13 *(dd, J* 7 = 7.9, 9.2)	74.7
3	3.35 (*dd, J* 7 = 9.2, 8.9)	78.1 a)	3.35 (*dd, J* 7 = 9.2, 8.9)	78.0^b^))
4	3.29 *(dd, J* 7 = 8.9, 9.7)	71.6	3.29 *(dd, J* 7=8.9, 9.7)	71.6
5	3.31 (*dd, J* 7 = 9.7,2.0,5.5)	78.0 a)	3.30 *(ddd.J =* 9.7, 2.2, 5.5)	78.0 b)
6a	3.88 (*dd, J* 7 = 2.0,11.9)	62.8	3.88 (*dd, J* = 11.9, 2.2)	62.8
6b	3.68 (*dd, J* 7 = 5.5,11.9)		3.68 (*dd, J* 7 = 11.9, 5.5)	
**r**	3.54(*t, J* 7 = 6.7)	62.9		182.9
2'	1.55(*tt, J* 7 = 7.8,6.7)	33.5	2.16(1,7=7.6)	39.2
3'	1.39(*tt, J* 7 = 7.5,7.8)	26.6	1.60-1.66 (m)	27.5
4'	1.51 (*tt, J* 7 = 7.5,7.5)	29.8	1.48-1.54 (m)	30.1
5'	2.23(*tdtd, J* 7 = 7.5, 6.8, 1.8, 1.0)	32.4	2.20-2.26 (m)	32.4
6'	6.12 (*ttd, J*.7 = 6.8, 2.7, 1.1)	124.1	6.12 (*ttd, J* 7=6.8, 2.7, 1.1)	124.2
7'		123.9		123.8
8'a	1.34 *(dddt, J* 7 = 10.1, 6.2, 2.7,1.8)	11.8	1.32-1.36 (m)	11.9
8'b	1.24 *(dddt, J* 7-10.1, 2.7, 2.7,1.8)		1.22-1.26 (m)	
9'	4.18 *(dddt, J* 7 = 6.2, 2.7, 1.1, 1.0)	52.7	4.16-4.18 (m)	52.8

a,b As, Assignments may be interchanged.

**Figure 3:**
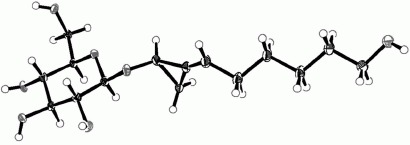
ORTEP Plot (ellipsoids 50% probability) of compound 1

Compound **1** additionally was crystallized in a solvent mixture of MeOH and Et_2_O. X-Ray-diffraction analysis established the relative configuration of the stereogenic centre in the methylidenecyclopropyl-alcohol moiety relative to glucose (Fig. 3)^1^) The absolute configuration of the glucose was established after hydrolysis, reaction of the sugar with chiral (−)-(*R*)-butan-2-ol, trimethylsilylation, and GC/MS analysis of the resulting derivative [5] [6], and, consequently, the absolute configuration of the aglycone was deduced as (*R*).

The ESI-MS of compound **2** displayed the [*M* − H]^−^ion peak at *m*/*z* 331 in the negative-ion mode , and the [*M*+H]+, [*M*+NH_4_]+, and the [*M*+Na]+ ion peaks at *m*/ *z* 333, 350, and 355, respectively, in positive-ion mode, providing a molecular weight of 332 Da. Due to the presence of the carboxylic acid group in 2, stable Y^−^_0_ and Z^−^_0_ fragment ions (*m*/*z* 169 and 151, resp.) could be detected upon MS/MS of the [*M* − H]^−^ ion. In addition, peaks of the cross-ring fragment ions ^0,2^A^−^ and ^0,3^A^−^ at *m*/*z* 119 and 89, respecively, were observed again. The MS/MS measurements in the positive-ion mode revealed corresponding results.

The NMR data of compound **2** were very similar to those of compound **1**. Again the sugar part was a β-glucopyranose with the glycosidic bond to the unusual methylidenecyclopropane alcohol. Differences were detected in the side chain: the signals for the terminal CH_2_ − OH group were missing, one CH_2_ signal was shifted to lower field, and a CO group signal at 182.9 ppm appeared in the ^13^C-NMR spectrum showing a HMBC to the afore mentioned CH_2_ group. Accordingly, the terminal alcohol in the alkane side chain of **2** should have been oxidized in **2** to give the corresponding carboxylic acid, (6*E*)-6-[2-(β-glucopyranosyloxy)cyclopropylidene]hexanoic acid.

Both compounds have, to the best of our knowledge, never been described in the literature, neither isolated from natural sources nor synthesized. The common characteristic structural feature of these new glycosidic compounds is the extremly rare methylidenecyclopropane alcohol as the aglycon moiety.

β-Glucopyranose was also determined as the sugar part in compounds **3** and **4**, but their aglycons were different. Both genins are phenolic acids for which differing NMR signals in the aromatic region were observed: the ^1^H-NMR spin system for a trisubstituted benzene together with the (*E*)-C = C bond revealed compound **3** as caffeic acid. On the other hand, compound **4**, showing only two *doublet* signals, could be identified as (*E*)-*p*-coumaric acid. HMBC Cross-peaks established the glycosidic linkage to the phenol in the opposite position of the conjugated acid in both compounds. On the basis of these data, **3** was identified as 4-*O*-β-d-glucopyranosylcaffeic acid and **4** as (*E*)-4-*O*-β-d-glucopyranosylcoumaric acid (Fig. 1). Their NMR spectroscopic data corresponded to those published [7]. Compounds **3** and **4** are not very widespread in plant kingdom. They have been isolated from several ferns such as *Davillia mariesii* Moore, *Camptosorus sibiricus* Rupr., and *Matteuccia struthiopteris* (L.) Tod. [7 – 9]. Additionally, 4-*O*-β-d-glucopyranosylcaffeic acid (**3**) has been found in the Colchicaceae family [10] and (*E*)-4-*O*-β-d-glucopyranosylcoumaric acid (**4**) in Fabaceae [11] [12], and both only in few other plant species so far.

Compound **5** was identified as a methyl glycoside. Two CH_2_groups and one quarternary C-atom indicated a ketose. The detailed NMR analysis and comparison of the ^13^C-NMR data with those in the literature [13] revealed compound **5** as methyl afructofuranoside.

The new compounds **2** and **2** were tested for *in vitro* cytotoxic activity against the colon carcinoma cell line SW480. Cell viability was assessed by a neutral red uptake assay as described in [14]. The compounds did not exhibit significant activity (*IC*_50_ 250 μm).

In conclusion, the chemical analysis of the Costa Rican tree fern *Metaxya rostrata* C.Presl yielded two new compounds, (1*R*,2*E*)-2-(6-hydroxyhexylidene)cyclopropyl β-D- glucopyranoside (**1**) and (6*E*)-6-[(2*R*)-2-(β-glucopyranosyloxy)cyclopropylidene]- hexanoic acid (**2**). Thus, for the very first time glucosides of a new natural methylidenecyclopropane alcohol were isolated. To the best of our knowledge, this methylidenecyclopropane alcohol has also never been synthezised before. In addition, two phenolic acid glycosides, 4-*O*-β-d-glucopyranosylcaffeic acid (**3**) and (*E*)-4-*O*-β-dglucopyranosylcoumaric acid (**4**), and methyl a-fructofuranoside (5) were isolated for the first time from this plant.

## Experimental Part

*General*. Anal.-grade solvents for extraction and fractionation were obtained from VWR (AVienna). TLC: on silica gel plates (*Merck*, Germany), mobile phase AcOEt/HCOOH/MeOH/H_2_O 70:8:8:11, and detection with anisaldehyde/sulfuric acid reagent [15]. Optical rotation: *Perkin Elmer Polarimeter 341*. UV/VIS Spectra: *Beckman DU640* spectrophotometer. IR Spectra: *Perkin Elmer FT-IR 2000* instrument in attenuated total reflection mode using a *Golden Gate* ATR unit. All NMR spectra were recorded on a *Bruker Avance DRX 600* NMR spectrometer using a 5 mm switchable quadruple probe (QNP, ^1^H, ^13^C, ^19^F, ^31^P) with z axis gradients, and automatic tuning and matching accessory; ^1^H: 600.13 and ^13^C: 150.92 MHz. All recordings were performed in a soln. in CD_3_OD at 298 K. Standard 1D and gradient-enhanced (ge) 2D experiments such as double quantum filtered (DQF) COSY, TOCSY, NOESY, HSQC, and HMBC were conducted as instructed by the manufacturer. Chemical shifts are referenced internally to the residual, non-deuterated solvent signal for ^1^H (δ 3.31 ppm) or to the C-atom signal of the solvent for 13C (δ 49.00 ppm). The analysis of the ^1^H,^1^H coupling constants (in Hz) were supported by the program Spin-Works (provided by *Kirk Marat*, University of Manitoba, Canada). ESIMS Data were acquired on a *API* 4000 triple quadrupole mass spectrometer (*AB Sciex Instruments*, Foster City, CA, USA) in both negative- and positive-ionization mode. Mass spectra were recorded over the range *m/z* 100 – 1000; scan time was 1 s. Product ions (MS/MS) were scanned from *m/z* 40 – 400 within 1 s of scan time. FIA was carried out with MeOH/aq. NH4OAc 5 mmol (80:20). HR-ESI-MS Data were obtained with a *Biosystems QStar* (Q-TOF) instrument in ESI negative-ion mode.

*Crystallographic Structure Determination*. X-Ray diffraction measurement was performed on a *Bruker X8 APEX II CCD diffractometer*. Single crystal of 1 was mounted on glass fiber, coated with Parathone N oil and positioned at 35 mmfrom the detector. 890 frames were measured, each for 50 s over 18 scan width. The data were processed using SAINT software [16]. Crystal data, data collection parameters, and structure refinement details are compiled in Table 2. The structure was solved by direct methods and refined by full-matrix least-squares techniques. The non-H-atoms were refined with anisotropic displacement parameters, while the H-atoms were placed at geometrically calculated positions and refined as riding atoms in the subsequent least-squares model refinements. The isotropic thermal parameters were estimated to be 1.2 times the values of the equivalent isotropic thermal parameters of the atoms to which H-atoms were bonded. The following software programs and computer were used: structure solution, SHELXS-97 [17]; refinement, SHELXL-97 [18]; molecular diagrams, ORTEP-3 [19]; computer, *Intel CoreDuo*.

**Table 2:** X-Ray Crystallographic Data for.

Empirical formula	C_15_,H_26_,O_7_
Formula weight	318.36
Temp. [K]	100(2)
Crystal system, space group	monoclinic, *P*2_1_
Unit cell dimensions *a* [Å]	9.5033(10)
*b* [Å]	8.4361(13)
c [Å]	20.625(3)
β [°]	99.362(4)
*V* [Å^3^]	1631.5(4)
Z	4
Calc. density [mg/mm^3^]	1.296
Absorption coefficient [mm^−1^]	0.102
*F*(000)	688
Crystal size [mm]	0.30 × 0.15 × 0.05
*θ* Range [°]	2.24-28.00
Index ranges	−11 ≤ *h* ≤ 12
	−27≤A≤27
Reflections collected	22480
Reflections unique	4039
R(int)	0.0748
Completeness to *2D =* 56.00	96.1%
Data/restraints/parameters	4039/1/407
Goodness-of-fit on *F^2^*	1.025
Final R[*I*>2σ(*I*),*w*R2	0.0472,0.1150
Largest diff. peak/hole [e Å^−3^]	0.314/−0.226

*Plant Material. Rhizomes of Metaxya rostrata* C.Presl were collected in February 2003 in the surroundings of La Gamba, south-western Costa Rica, and authenticated in the Herbarium of the Museo National in San Jose by *Dr. Werner Huber*. Voucher specimens (Voucher number: MR0203) are deposited with the Herbarium of the Department of Pharmacognosy, University of Vienna, Austria.

*Extraction and Isolation*. Dried roots (800 g) of *Metaxya rostrata* C.Presl were pulverized and portions of 100 g each were extracted by sonification at 50° with 1 l H_2_O, each. The extraction was repeated five times with the same amount of solvent. After lyophilization, the residue (80 g) was extracted sequentially with AcOEt, BuOH, and MeOH. The fractions of different polarity (AcOEt fraction (0.75 g), BuOH fraction (26.35 g), and MeOHfraction (18.7 g)) were subjected to vacuum liquid chromatography (VLC) on silica (15 – 40 μm, *Merck*) with AcOEt/MeOH/H_2_O mixtures of increasing polarity as mobile phases. All obtained fractions after these three fractionation steps were pooled according to their composition to 15 combined fractions [1]. *Fr. 12 (2.96 g)* was chosen for further separation in this study and subjected to column chromatography (CC) on Sephadex LH-20 (3 − 65 cm) with 80% MeOH. Of 13 subfractions, *Frs. 12/3 (0.50 g), 12/4 (0.69 g), and 12/5 (0.55 g)* were further fractionated.

*Fr. 12/3* was subjected to CC (silica gel *60* (particle size 0.063 – 0.200 mm; Merck; H_2_O-sat. AcOEt/ MeOH 90:10) to yield 13 subfractions. From Subfr. 12/3/3, compound **2** (58 mg) crystallized. Subfr. 12/3/ 10 was further purified by CC (*Sephadex LH-20* (1 × 40 cm); 50% MeOH) to give 3 mg of compound **2** and Subfr. 12/3/2 by CC (silica gel 60; H_2_O-sat. AcOEt) to afford 7 mg of compound **5**.

*Fr. 12/5* was subjected to CC (silica gel 60; H_2_O-sat. AcOEt/MeOH 90:10) to yield nine subfractions. Subfr. 12/5/9 was further purified by CC (silica gel 60; H_2_O-sat. AcOEt/MeOH 80:20) and resulted in the isolation of compound **3** (12 mg).

*Fr. 12/4* was subjected to CC (silica gel 60; H_2_O-sat. AcOEt/MeOH 90 :10) to give compound **4** (1.2 mg).

*Sugar Analysis*. The determination of the absolute configuration of the sugar was achieved after enzymatic hydrolysis of 1 mg of compound **1** with β-glucuronidase (No. G0751 from Helix pomatia; Sigma-Aldrich, A-Vienna), butylation with (−)-(R)-butan-2-ol, and derivatization with N-methyl-N- (trimethylsilyl)trifluoracetamide according to [5] [6]. GC/MS Analyses were performed on a GCMS-QP 2010 (Shimadzu Corporation, Kyoto, Japan) according to [6].

(1R,2E)-2-(6-Hydroxyhexylidene)cyclopropyl β-d-Glucopyranoside (**1**). Colorless needles. M.p.: 129 – 131°. [α]20 D = − 74.9 (c = 0.41, MeOH). IR (neat): 3364, 3035, 1775, 1169, 1107, 1063, 1034. ^1^H- and 13C-NMR: see Table 1. ESI-MS (pos.): 319 ([M+H]+), 336 ([M+NH4]+), 341 ([M+Na]+), 157 (Y^+^_0_ ), 139 (Z^+^_0_ ). ESI-MS (neg.): 317 ([M − H]^−^), 179 (C^−^_1_ ), 161 (B^−^_1_ ), 119 (0,2A^−^), 89 (0,3A^−^). HR-ESI-MS: 317.3539 ([*M* − H] ^−^, C15H25O^−^_7_ calc. 317.1600).

*(6E)-6-[(2R)-2-(β-d-Glucopyranosyloxy)cyclopropylidene]hexanoic Acid* (**2**). Colorless, amorphous powder. [α]^20^_D_ = −69.3 (c = 0.32, MeOH). ^1^H- and 13C-NMR: see Table 1. ESI-MS (pos.): 333 ([*M*+H]+), 350 ([*M*+NH4]+), 355 ([*M*+Na]+). ESI-MS (neg.): 331 ([M − H]^−^), 169 (Y^−^_0_), 151 (Z^−^_0_ ), 119 (0,2A^−^), 89 (0,3A^−^). HR-ESI-MS: 331.3378 ([M − H]^−^, C15H25O^−^_7_ ; calc. 331.1600).

*Cytotoxicity Assay*. SW480 Carcinoma cells obtained from the American Type Culture Collection and cultivated under standard conditions (minimal essential medium (MEM) containing 10% fetal calf serum (FCS)) were used to assess the cytotoxic capacity of compounds **1** and **2**. Cell viability was assessed by neutral red uptake assay [14].

Financial support of the FWF (project-No. P20354) is gratefully acknowledged.We thank *Alexander Roller* (University of Vienna, Institute of Inorganic Chemistry) for single-crystal X-ray diffraction measurements.
